# Pseudoaneurysm Following Stereotactic Breast Biopsy

**DOI:** 10.7759/cureus.115514

**Published:** 2026-08-31

**Authors:** James D Fox, Ahmed Abdalla, Kirubahara Vaheesan, Paula George

**Affiliations:** 1 Radiology, Saint Louis University School of Medicine, Saint Louis, USA; 2 Interventional Radiology, Stl Interventional Oncology Interventional Radiology Clinics, LLC, Creve Coeur, USA; 3 Radiology, SSM Saint Louis University Hospital, Saint Louis, USA

**Keywords:** breast cancer imaging, breast lesions and imaging modality, embolization, interventional radiology, interventional radiology guided embolization, mammography, microcalcifications, pseudoaneurysm, stereotactic breast biopsy, thrombin injection

## Abstract

Stereotactic breast biopsy is a widely utilized and generally low-risk method for evaluating suspicious breast findings, though rare vascular complications, such as pseudoaneurysm, can occur. This case describes a patient who developed a delayed pseudoaneurysm approximately two weeks after an otherwise routine biopsy with benign pathology. The patient initially developed a post-procedural hematoma and subsequently presented with worsening pain, prompting further evaluation. Additional imaging revealed a pseudoaneurysm. Although first-line treatment with ultrasound-guided thrombin injection was attempted, recurrence necessitated definitive endovascular embolization, which successfully resolved the condition. This case highlights the potential for delayed presentation, the importance of considering vascular injury in patients with persistent symptoms, and the need for prompt imaging and coordinated multidisciplinary care to ensure accurate diagnosis and effective, stepwise management.

## Introduction

Stereotactic breast biopsy is a widely used, low-risk, and well-established procedure for sampling breast lesions, particularly microcalcifications detected on mammography that lack a sonographic correlate. Most of these procedures are performed without incident. However, post-procedural pseudoaneurysms are a known rare but potentially serious complication of needle core breast biopsies [1-3]. A pseudoaneurysm or false aneurysm can occur when there is a disruption of the arterial wall, resulting in an extravascular blood collection contained only by a surrounding hematoma, and not the three layers of an arterial wall. In the breast, pseudoaneurysms typically present following a biopsy with a painful, pulsatile mass at or near the biopsy site. On ultrasound, there is blood flow within a mass or hematoma, with the characteristic "yin-yang" sign [4]. In this article, we present a case of a breast pseudoaneurysm arising from the left thoracodorsal artery following a stereotactic biopsy performed for microcalcifications. The pseudoaneurysm was initially treated with thrombin injection under ultrasound guidance; after recurrence, subsequent coil embolization was performed. This case highlights the importance of early sonographic recognition and a multidisciplinary approach to potential vascular injuries resulting from image-guided breast biopsies.

The incidence of pseudoaneurysm following breast biopsy has not been precisely quantified in the literature. Pseudoaneurysm is described as a rare complication, with only isolated case reports and small case series published over the past two decades despite the widespread use of core needle and vacuum-assisted breast biopsy techniques [5-8].

## Case presentation

A 68-year-old female with a past medical history of diabetes and tobacco use and no family or personal history of breast cancer presented for a diagnostic bilateral mammogram for left breast pain, which demonstrated a new small group of coarse heterogeneous microcalcifications in the upper outer quadrant of the left breast (Figures 1A-1B). Pre-procedure blood pressure was not taken, but prior blood pressures were normotensive. A stereotactic breast biopsy was performed via a lateral approach, using a 9-gauge Hologic Eviva vacuum-assisted incisional core biopsy needle. Several samples were obtained with microcalcifications identified in both the specimen radiographs and on pathological examination. The procedure lasted approximately 30 minutes. Post-procedure mammogram obtained after standard manual compression over the biopsy site by the nurse for 20 minutes demonstrated a 4.6 cm hematoma at the biopsy site (Figure 2). The patient was not on any anticoagulation or antiplatelet medications and had no history of coagulopathy. She was asymptomatic at this time and managed by applying site compression with an all-cotton elastic (ACE) compression bandage wrapped around her chest, as well as instructions to place an ice pack over the biopsy site. She was also instructed to keep this bandage on for the next 48 hours and to call our office if she developed pain or the lump enlarged. The pathological results of the biopsy revealed benign breast tissue with fibrosis and microcalcifications (Breast Imaging Reporting and Data System category 2, BI-RADS 2).

**Figure 1:**
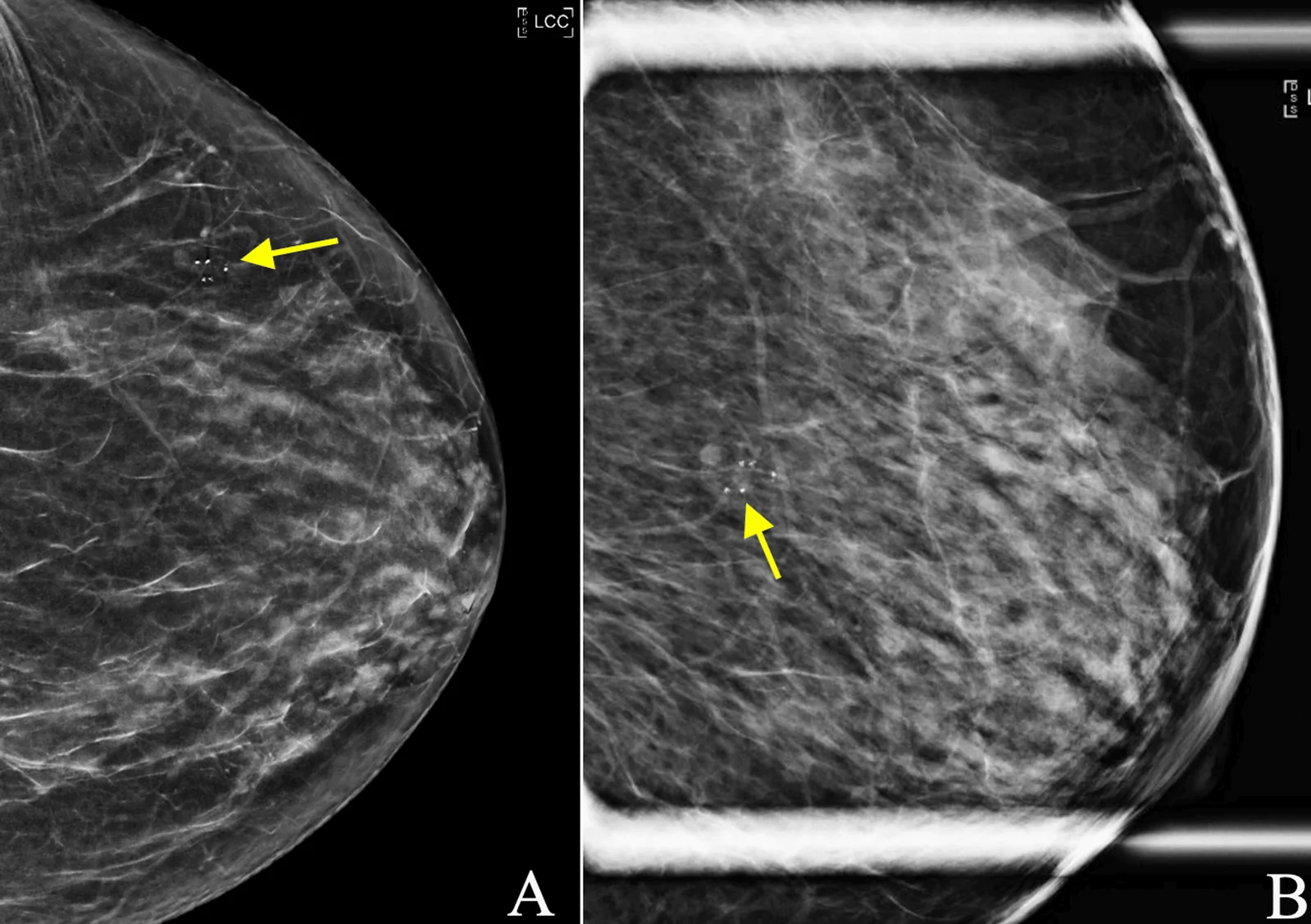
Diagnostic left craniocaudal (LCC) view (A) Mammogram view demonstrates a small group of coarse heterogeneous microcalcifications in the lateral left breast (solid yellow arrow). (B) Spot magnification compression true lateral view confirms a small group of microcalcifications (solid yellow arrow).

**Figure 2:**
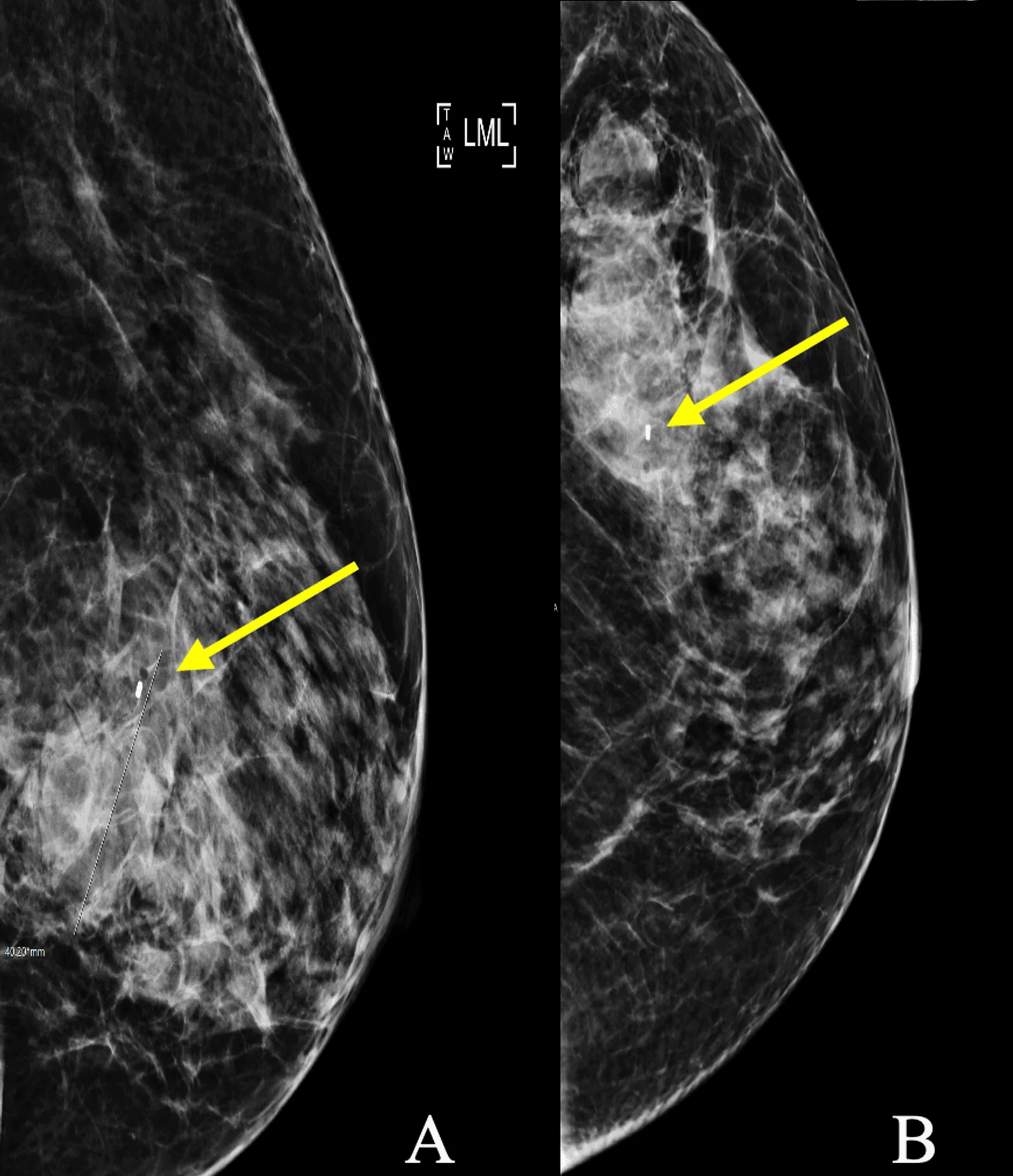
Left mediolateral (LML) (A) and left craniocaudal (LCC) (B) post-biopsy mammographic views These demonstrate a 4.6-cm hematoma at the biopsy site. Note the minicork tissue marker at the biopsy site (arrows).

The patient presented to the ED two days after the biopsy for left breast pain. It was noted in the ED that the patient had not been wearing the ACE bandage since the biopsy, and the patient had been drinking alcohol that day. Bedside ultrasound by the ED staff demonstrated a hematoma at the biopsy site similar in size to the post-biopsy mammogram. She was then placed in an ACE compression bandage and instructed to follow up with the breast imaging department or to return to the ED with worsening breast pain.

Two weeks following the biopsy, the patient presented to the breast imaging department with significant worsening pain in her left breast. A limited ultrasound of the left breast performed at our department demonstrated a 4.6 cm hematoma at the biopsy site, with central color flow, indicating a pseudoaneurysm (Figure 3). There was no significant interval change in the size of the hematoma from prior post-biopsy imaging performed on the day of the biopsy. Interventional radiology (IR) was consulted at that time for thrombin injection of the pseudoaneurysm. Computed tomography angiogram of the chest with intravenous contrast was also performed two weeks after the biopsy and revealed a 5.0 cm hematoma containing a 1.8 cm pseudoaneurysm in the upper outer quadrant of the left breast at the prior biopsy site (Figure 4). IR then advanced a 21-gauge coaxial needle under ultrasound guidance into the pseudoaneurysm and injected 500 units of diluted thrombin (Figure 5). There was complete thrombosis of the pseudoaneurysm. One-week follow-up left breast ultrasound was recommended.

**Figure 3:**
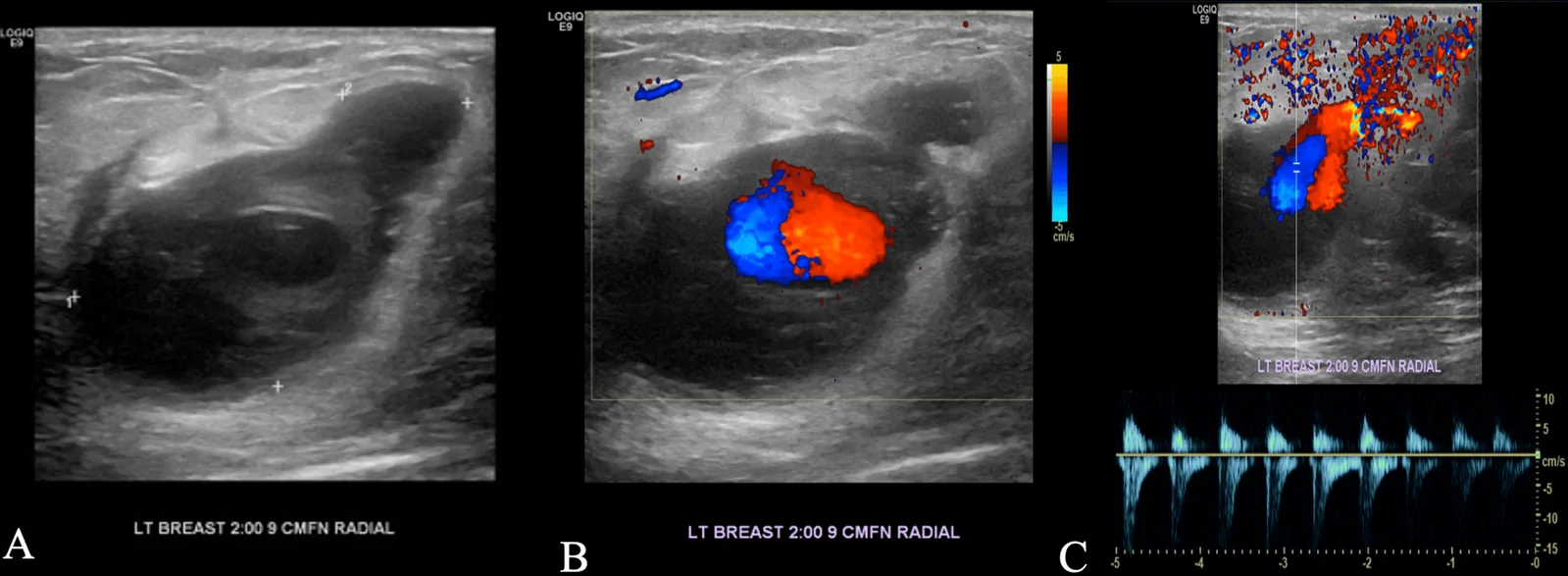
Ultrasound of the left breast (A) Ultrasound of the left breast 2:00 region demonstrating a 4.6 cm heterogeneously echogenic mass with the central anechoic area. (B) Ultrasound color Doppler of the same area demonstrated blood flow centrally within the mass with the typical "yin-yang" appearance of a pseudoaneurysm. (C) Spectral Doppler of the same mass demonstrated the typical pseudoaneurysm "to-and-fro" pattern

**Figure 4:**
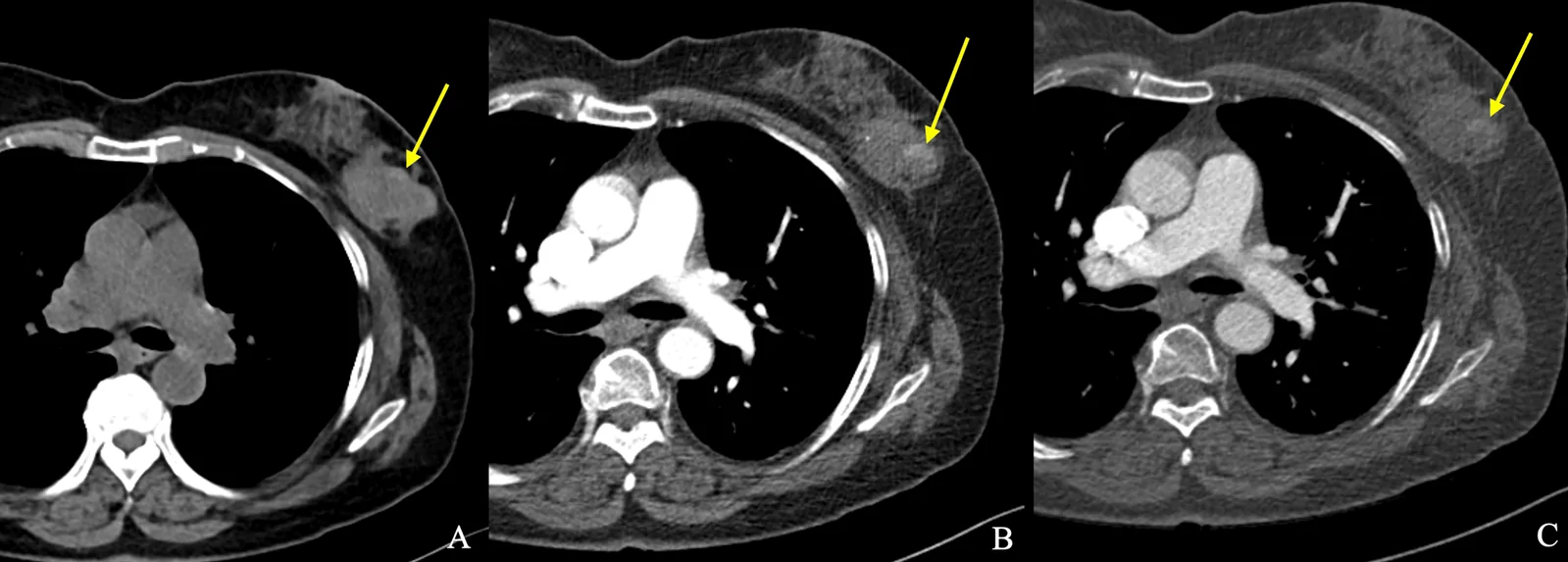
CT angiogram of the chest at the level of the pulmonary artery (A) Pre-contrast phase, (B) arterial phase, and (C) venous phase demonstrating a 5.0 cm hematoma in the upper outer quadrant of the left breast at the prior biopsy site (arrow) Within the lateral margin of the hematoma, there is a 1.8 cm hyperdensity that appears in the arterial phase and washes out in the venous phase, denoting a site of pseudoaneurysm.

**Figure 5:**
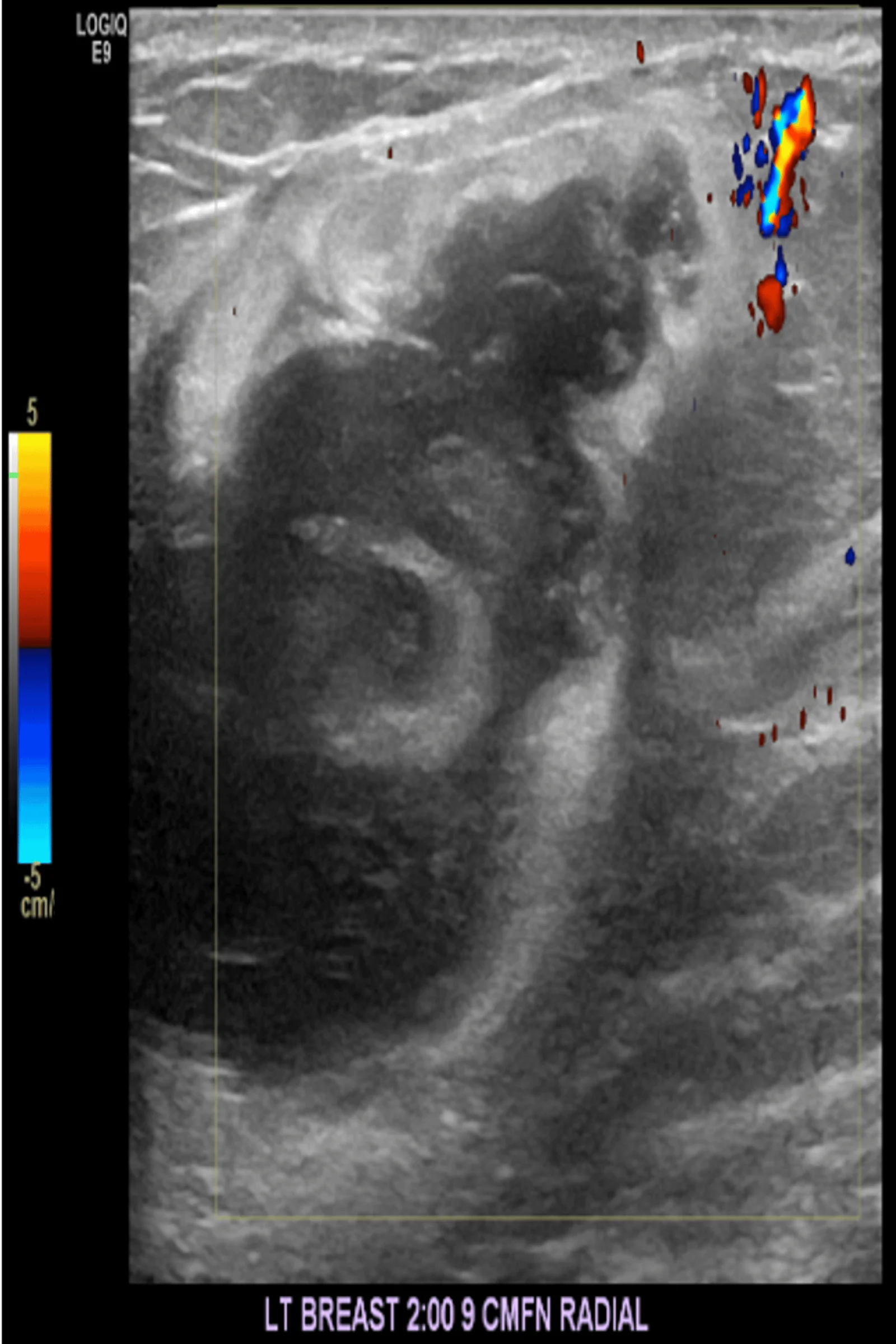
Ultrasound image of the left breast immediately post-thrombin injection

Six days after the thrombin injection, the patient developed an acute onset of marked pain and swelling in her left breast following a bus ride. She had not had any pain up to that point since the thrombin injection. An ultrasound was then performed of the left breast and demonstrated a recurrent 4.9 cm hematoma with a central pseudoaneurysm in the same region (2:00) of the left breast (Figure 6). IR was re-consulted for embolization of the pseudoaneurysm.

**Figure 6:**
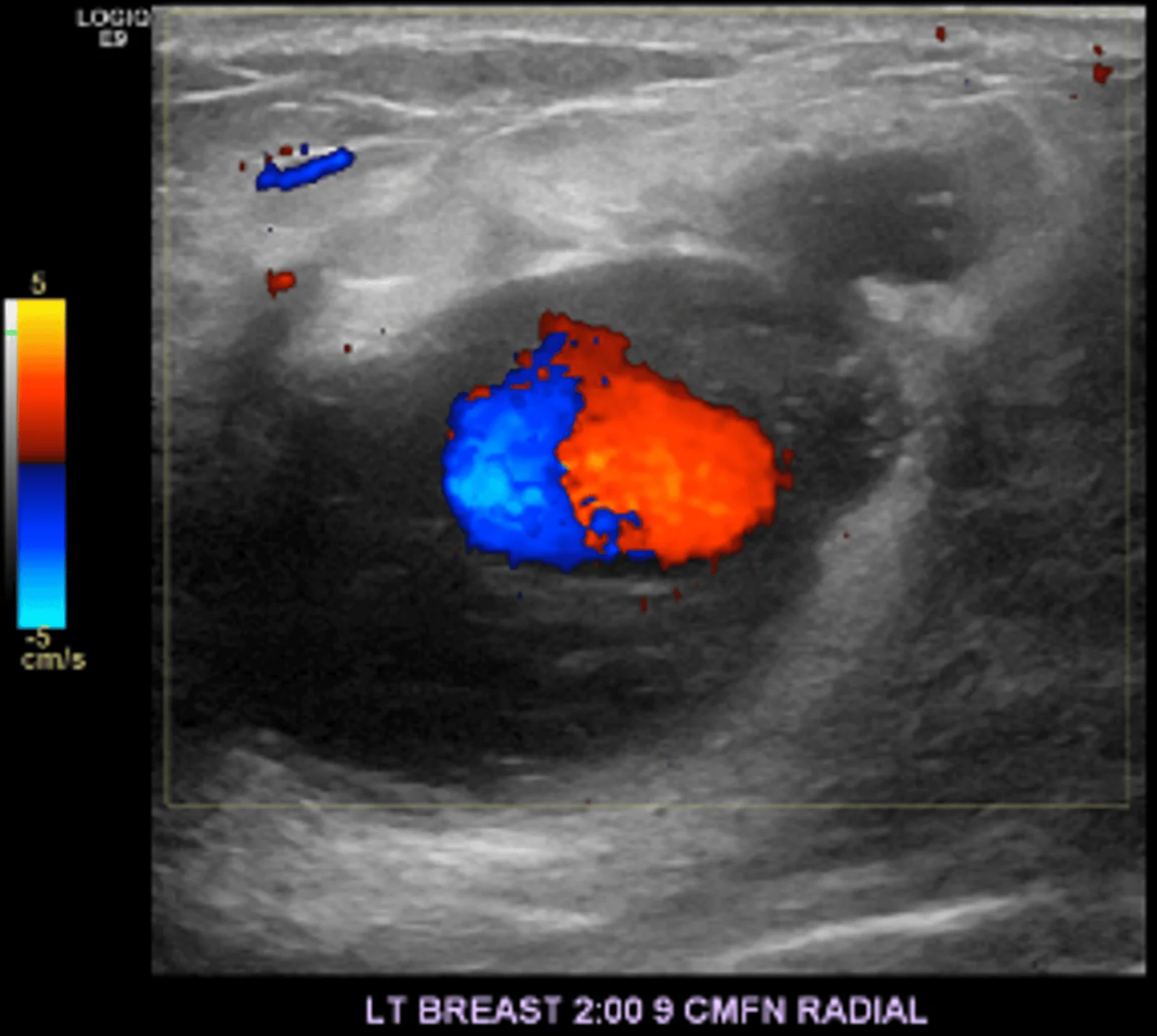
Ultrasound of the left breast Recurrent 4.9 cm hematoma with a central pseudoaneurysm one week after thrombin injection.

Embolization was performed through ultrasound-guided access via the patient's left radial artery. The left axillary, lateral thoracic, and thoracodorsal arteries were all selectively catheterized with subsequent angiograms. The pseudoaneurysm was discovered to arise from the left thoracodorsal artery, which was then embolized with two 2 mm x 4 cm coils (Figure 7).

**Figure 7:**
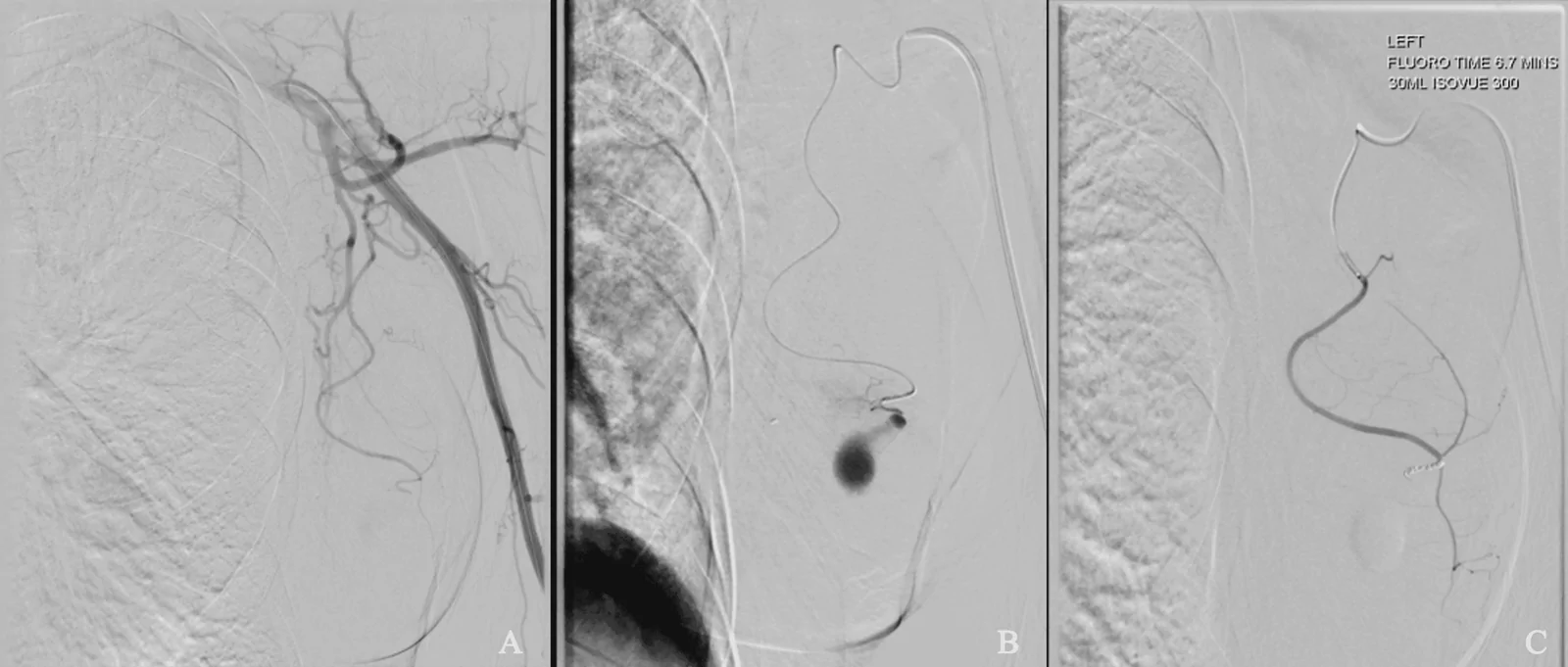
Digital subtraction angiography (A) Left lateral thoracic artery and branches, (B) large pseudoaneurysm arising from the left thoracodorsal artery, and (C) post-coil embolization demonstrates complete occlusion of the pseudoaneurysm.

Follow-up limited left breast ultrasound performed six days after the embolization demonstrated resolution of the previously noted pseudoaneurysm, with no significant change in size of the hematoma (Figure 8). The patient was asymptomatic at that time. She followed up with IR seven days later via a video visit and was then released from their service. She was instructed to resume annual screening mammograms, pending no interval breast concerns. The patient has remained asymptomatic since that time.

**Figure 8:**
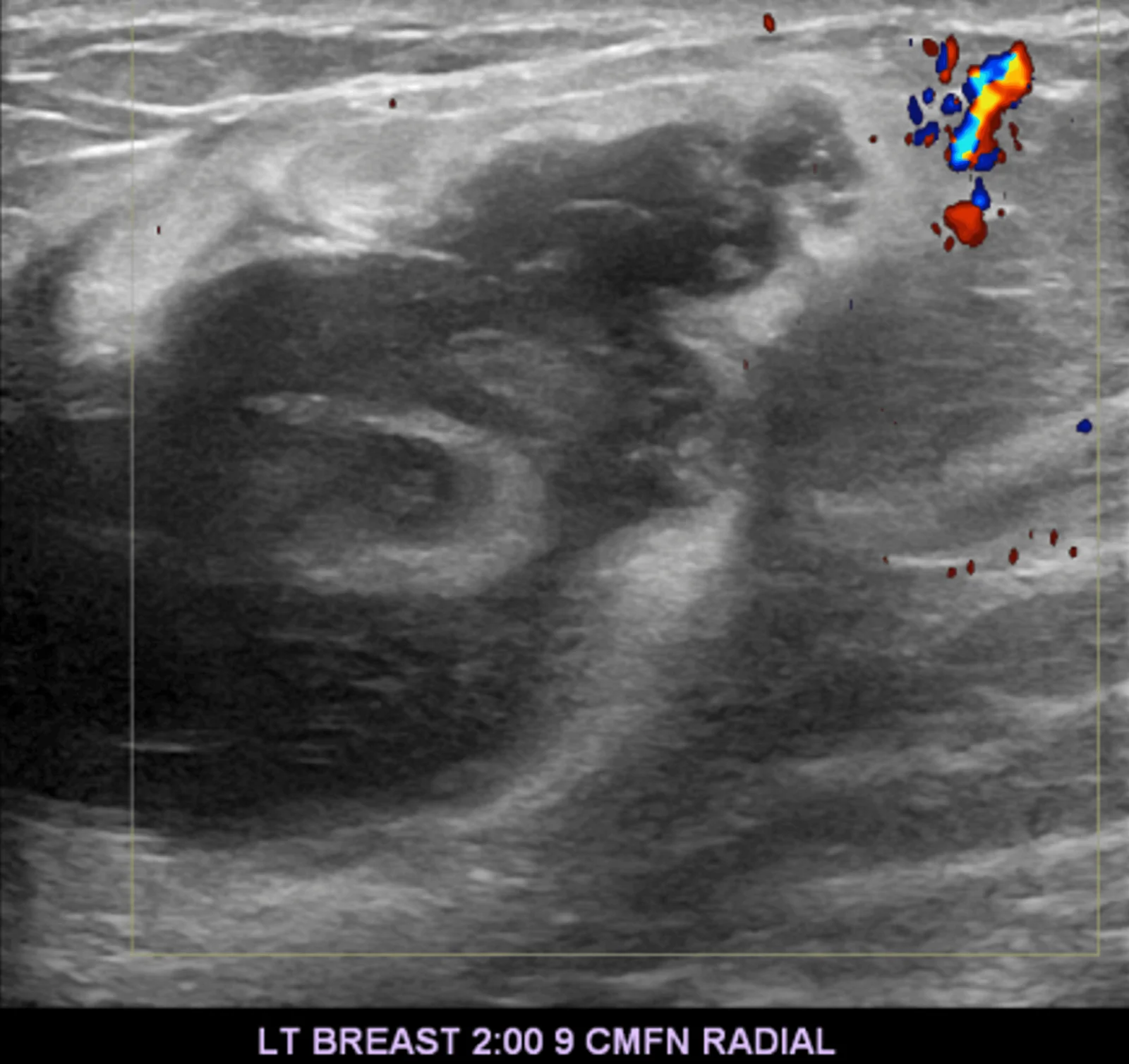
Post-embolization color Doppler ultrasound exam performed six days following the coiling procedure demonstrates no internal vascularity within the hematoma and resolved pseudoaneurysm

## Discussion

Vascular injury following percutaneous breast biopsy remains an infrequent but clinically significant complication, with the published experience consisting predominantly of isolated case reports and small institutional series [5,7,9]. When pseudoaneurysms arise in this setting, they most commonly originate from branches of the lateral thoracic or internal mammary arteries [5,10]. Patients typically present in a delayed fashion - often one to three weeks after the index procedure - with progressive pain, swelling, or a palpable pulsatile mass [5,7]. The present case aligns with this pattern, manifesting approximately two weeks following stereotactic vacuum-assisted biopsy. Additionally, 9-gauge needles are considered the standard of care for stereotactic breast biopsy for microcalcifications, in order to obtain adequate samples for pathologic diagnosis [11,12].

Sonographic evaluation with color and spectral Doppler remains the primary diagnostic modality, demonstrating the pathognomonic "yin-yang" sign - swirling bidirectional flow within the sac - and a to-and-fro waveform at the neck connecting the pseudoaneurysm to the parent artery [5,13,14]. These findings reliably distinguish pseudoaneurysms from simple hematomas or other cystic lesions [15,16]. However, noninvasive imaging may not definitively delineate the precise feeding vessel, particularly when complex vascular anatomy or atypical arterial origins are involved. In such scenarios, catheter-based angiography is needed for accurate vessel mapping and procedural planning, as illustrated in this case where the thoracodorsal artery was definitively identified only at the time of embolization.

Ultrasound-guided percutaneous thrombin injection has emerged as the preferred first-line therapy for iatrogenic pseudoaneurysms, with reported success rates ranging from 93% to 97% in large series [17,18]. The technique is minimally invasive and well-tolerated and obviates the need for an interventional procedure in most patients. Nonetheless, recurrence occurs in approximately 5% of cases, and certain anatomic or morphologic features - including larger pseudoaneurysm size, complex neck geometry, or high-flow states - may predispose to treatment failure [18,19]. On color Doppler, the pseudoaneurysm neck was 0.3 cm, which would not be considered a large neck and therefore did not represent an obvious anatomic risk factor for treatment failure. When thrombin injection proves insufficient, endovascular coil embolization of the feeding artery offers a definitive alternative [5,20]. This escalation was warranted in the present case following symptomatic recurrence, underscoring the importance of a flexible, stepwise management algorithm.

This case also prompted several changes in clinical practice within our institution. In retrospect, an ultrasound with Doppler after the procedure may have detected a pseudoaneurysm earlier. However, the patient had no pain immediately after the procedure, so an ultrasound was not performed. The patient initially presented to the ED in the evening and was instructed to follow up with the breast center. The referral workflow between the ED and breast imaging department has since improved to expedite follow-up care for future patients.

Ultimately, a pseudoaneurysm following a stereotactic breast biopsy is a known risk of the procedure. This risk can be significantly mitigated with proper post-biopsy care. In this case, nonadherence to the recommended ACE compression bandage most likely contributed to the persistence or progression of the pseudoaneurysm.

## Conclusions

Overall, this case highlights the need for heightened clinical awareness of this rare complication and the importance of considering vascular injury in patients with persistent or worsening breast pain following a biopsy. Multimodality imaging and coordinated multidisciplinary care remain essential for accurate diagnosis and effective management.

Radiologists performing or interpreting breast biopsies should be aware of this rare but potentially serious complication, particularly when patients report delayed-onset pain or swelling. Prompt ultrasound with Doppler evaluation, multidisciplinary consultation involving interventional radiology, and structured follow-up imaging are essential to ensure timely diagnosis and appropriate therapeutic escalation when first-line measures prove inadequate.

## References

[1] Binh NT, Duc NM, Tra My TT, Dung LV, Luu DT, Linh LT (2021). Emergency embolization for breast pseudoaneurysm following vacuum-assisted biopsy: a case report. Pan Afr Med J.

[2] Gadsbøll E, Warming N, Kragh EL, Juhl AA (2024). Acute pseudoaneurysm following cutaneous carcinoma excision: management and clinical insights. BMJ Case Rep.

[3] Jayathilake C, Uyangoda T, Kitsos D, Sever A, Kasem A, Metafa A (2024). Two cases of breast pseudoaneurysm following core biopsy: a very rare complication with different treatment options. Ultrasound.

[4] Pluguez-Turull C, Del Toro C, Brofman N, Feliciano YZ (2024). Pseudoaneurysm in the axillary tail of the breast after a core needle biopsy. Eur J Breast Health.

[5] Adler K, Samreen N, Glazebrook KN, Bhatt AA (2020). Imaging features and treatment options for breast pseudoaneurysms after biopsy: a case-based pictorial review. J Ultrasound Med.

[6] Sun S, Hennessey H, Kam Nakch I, Alsharif S, Meterissian S, Mesurolle B (2014). Compression-refractory breast hematoma secondary to pseudoaneurysm after stereotactically guided vacuum-assisted biopsy: the critical role of urgent surgical evacuation. J Clin Ultrasound.

[7] Swain B, Castelhano R, Litton K, Chaudhry A (2022). Core needle biopsy causing a pseudoaneurysm in the breast. Ann R Coll Surg Engl.

[8] Vignoli C, Bicchierai G, Masciotra MC (2019). Axillary pseudoaneurysm post core needle biopsy. Breast J.

[9] Dixon AM, Enion DS (2004). Pseudoaneurysm of the breast: case study and review of literature. Br J Radiol.

[10] Azorín Samper MD, Hernandorena González M, Ibáñez Arias A, Díaz Sierra S, Bertolo Domínguez M, Checa Ayet F (2019). A case report of pseudoaneurysm after a core needle biopsy of breast cancer. Breast J.

[11] Lourenco AP, Mainiero MB, Lazarus E, Giri D, Schepps B (2007). Stereotactic breast biopsy: comparison of histologic underestimation rates with 11- and 9-gauge vacuum-assisted breast biopsy. AJR Am J Roentgenol.

[12] Schaefer FK, Order BM, Eckmann-Scholz C (2012). Interventional bleeding, hematoma and scar-formation after vacuum-biopsy under stereotactic guidance: Mammotome(®)-system 11 g/8 g vs. ATEC(®)-system 12 g/9 g. Eur J Radiol.

[13] Vegas A, Wells B, Braum P (2025). Guidelines for performing ultrasound-guided vascular cannulation: recommendations of the American Society of Echocardiography. J Am Soc Echocardiogr.

[14] Habiyaremye TD, Alloy AC, Gibbons RC (2024). Finding the balance: the point-of-care ultrasound diagnosis of pseudoaneurysm. VDEM.

[15] Mitchell DG, Needleman L, Bezzi M (1987). Femoral artery pseudoaneurysm: diagnosis with conventional duplex and color Doppler US. Radiology.

[16] Rivera M, Villacorta J, Jiménez-Alvaro S, Quereda C (2011). Asymptomatic large extracapsular renal pseudoaneurysm following kidney transplant biopsy. Am J Kidney Dis.

[17] Kontopodis N, Tsetis D, Tavlas E, Dedes A, Ioannou CV (2016). Ultrasound guided compression versus ultrasound guided thrombin injection for the treatment of post-catheterization femoral pseudoaneurysms: systematic review and meta-analysis of comparative studies. Eur J Vasc Endovasc Surg.

[18] Hirsch AT, Haskal ZJ, Hertzer NR (2006). ACC/AHA 2005 Guidelines for the Management of Patients With Peripheral Arterial Disease (Lower Extremity, Renal, Mesenteric, and Abdominal Aortic): Executive Summary A Collaborative Report From the American Association for Vascular Surgery/Society for Vascular Surgery,⁎Society for Cardiovascular Angiography and Interventions, Society for Vascular Medicine and Biology, Society of Interventional Radiology, and the ACC/AHA Task Force on Practice Guidelines (Writing Committee to Develop Guidelines for the Management of Patients With Peripheral Arterial Disease). J Am Coll Cardiol.

[19] Esterson YB, Pellerito JS (2017). Recurrence of thrombin-injected pseudoaneurysms under ultrasound guidance: a 10-year retrospective analysis. J Ultrasound Med.

[20] Keeling AN, McGrath FP, Lee MJ (2009). Interventional radiology in the diagnosis, management, and follow-up of pseudoaneurysms. Cardiovasc Intervent Radiol.

